# Inflammatory and antimicrobial properties differ between vaginal *Lactobacillus* isolates from South African women with non-optimal versus optimal microbiota

**DOI:** 10.1038/s41598-020-62184-8

**Published:** 2020-04-10

**Authors:** Monalisa T. Manhanzva, Andrea G. Abrahams, Hoyam Gamieldien, Remy Froissart, Heather Jaspan, Shameem Z. Jaumdally, Shaun L. Barnabas, Smritee Dabee, Linda G. Bekker, Glenda Gray, Jo-Ann S. Passmore, Lindi Masson

**Affiliations:** 1 Institute of Infectious Disease and Molecular Medicine (IDM), University of Cape Town, Cape Town, South Africa; 2 UMR 5290 MIVEGEC, French National Centre for Scientific Research (CNRS), Montpellier, France; 3 Seattle Children’s Research Institute, University of Washington, Seattle, Washington USA; 4 Desmond Tutu HIV Centre, University of Cape Town, Cape Town, South Africa; 5 Perinatal HIV Research Unit, University of the Witwatersrand, Johannesburg, South Africa; 6 South African Medical Research Council, Cape Town, South Africa; 7 Centre for the AIDS Programme of Research in South Africa (CAPRISA), Durban, South Africa; 8 National Health Laboratory Service, Cape Town, South Africa; 9 Disease Elimination Program, Life Sciences Discipline, Burnet Institute, Melbourne, Australia

**Keywords:** Bacterial infection, Inflammation, Bacterial host response

## Abstract

Female genital tract (FGT) inflammation increases HIV infection susceptibility. Non-optimal cervicovaginal microbiota, characterized by depletion of *Lactobacillus* species and increased bacterial diversity, is associated with increased FGT cytokine production. *Lactobacillus* species may protect against HIV partly by reducing FGT inflammation. We isolated 80 lactobacilli from South African women with non-optimal (Nugent 4–10; n = 18) and optimal microbiota (Nugent 0–3; n = 14). Cytokine production by vaginal epithelial cells in response to lactobacilli in the presence and absence of *Gardnerella vaginalis* was measured using Luminex. Adhesion to vaginal epithelial cells, pH, D/L-lactate production and lactate dehydrogenase relative abundance were assessed. Lactobacilli from women with non-optimal produced less lactic acid and induced greater inflammatory cytokine production than those from women with optimal microbiota, with IL-6, IL-8, IL-1α, IL-1β and MIP-1α/β production significantly elevated. Overall, lactobacilli suppressed IL-6 (adjusted p < 0.001) and IL-8 (adjusted p = 0.0170) responses to *G. vaginalis*. Cytokine responses to the lactobacilli were inversely associated with lactobacilli adhesion to epithelial cells and D-lactate dehydrogenase relative abundance. Thus, while cervicovaginal lactobacilli reduced the production of the majority of inflammatory cytokines in response to *G. vaginalis*, isolates from women with non-optimal microbiota were more inflammatory and produced less lactic acid than isolates from women with optimal microbiota.

## Introduction

HIV remains a major public health concern, particularly in sub-Saharan Africa where young South African women are at an exceptionally high risk of becoming HIV-infected^1,2^. Increased production of inflammatory cytokines in the female genital tract (FGT) increases HIV acquisition risk, likely by recruiting activated HIV target cells, such as CD4+ T-cells, to the vaginal mucosal epithelium, promoting HIV transcription via nuclear factor kappa B (NF-κB) activation, and reducing the integrity of the epithelial barrier^3–6^. Bacterial vaginosis (BV) and non-optimal microbiota including *Gardnerella vaginalis*, *Prevotella bivia, Atopobium* spp., *Mycoplasma hominis* and *Mobiluncus* spp., are thought to be major drivers of FGT inflammation and HIV risk in sub-Saharan African women^7,8^. BV also increases susceptibility to other sexually transmitted infections (STIs) including *Chlamydia trachomatis*, *Neisseria gonorrhoeae, Trichomonas vaginalis*^9^, human papillomavirus^10^, herpes simplex virus type 2 (HSV-2)^11^, and adverse reproductive health outcomes^12^. Furthermore, HIV-infected women with BV are over 3-times more likely to transmit HIV to their partners^13,14^. However, the pathogenesis and immunomodulatory effects of BV are not yet fully understood and current treatment strategies are only partially effective, with inflammatory cytokine concentrations remaining elevated even in women who are successfully treated^15,16^.

On the other hand, optimal vaginal microbiota of healthy pre-menopausal women is dominated by *Lactobacillus* species, including *L. crispatus, L. jensenii, L. gasseri*, and *L. vaginalis*^17–19^*. Lactobacillus* species appear to play a critical role in regulating inflammatory responses in the FGT and protecting against pathogens, including HIV^5,20^. The role of *L. iners* is however controversial as this species is associated with increased risk of conversion from an optimal to a non-optimal vaginal microbiome^21^, acquisition of STIs^22^ and upregulation of inflammatory responses^23^. The mechanisms underlying the protective properties of non-*iners Lactobacillus* species that are considered to be optimal are not fully understood, however it is thought that lactobacilli protect against pathogens by competitively excluding pathogen colonization, and producing antimicrobial compounds such as bacteriocins and lactic acid^24^. Lactic acid exists as L- and D- isomers and it maintains a physiological pH of <4.5 in the FGT which inhibits the growth of potential pathogens and may inactivate HIV virus particles^25,26^. Competitive exclusion of pathogens by lactobacilli may modulate inflammation by preventing pathogen interaction with pattern recognition receptors (PRRs) present in the genital epithelium. Lactobacilli and the lactic acid that they produce may also downregulate inflammatory cytokine production by cervicovaginal epithelial cells, which may in turn reduce susceptibility to HIV^27,28^. A better understanding of the immunomodulatory and other properties of vaginal lactobacilli is critical for the development of biomedical interventions to improve BV treatment and reduce HIV infection risk in women. As few studies have characterized vaginal *Lactobacillus* isolates in African populations, we evaluated the influence of optimal vaginal *Lactobacillus* species isolated from South African women. This study included *Lactobacillus* species that are considered to be optimal and are associated with the lowest levels of inflammatory cytokine production *in vivo*, in order to further evaluate their immunomodulatory properties that may reduce HIV risk.

## Results

### Study population and clinical *Lactobacillus*  isolates

A total of 80 *Lactobacillus* isolates were obtained from the cervicovaginal secretions of 32 women who participated in the Women’s Initiative in Sexual Health (WISH) study in Cape Town, South Africa [*L. crispatus* (n = 15), *L. jensenii* (n = 18), *L. johnsonii* (n = 5), *L. mucosae* (n = 19), *L. plantarum* (n = 2), *L. ruminis* (n = 5), *L. salivarius* (n = 2), *L. vaginalis* (n = 14)]. BV status was determined by Nugent scoring (women with BV had Nugent scores ≥7; women with intermediate microbiota had Nugent scores between 4–6; women who were BV negative had scores between 0–3). We obtained 36 isolates from 18 women with non-optimal microbiota [intermediate microbiota (n = 5) and BV positive (n = 13)] and 44 isolates from 14 women with optimal microbiota [BV negative (n = 14)] (Table 1). The median age of the women was 18 (range 16–22) years and all of the women were using hormonal contraceptives at the time of sample collection. Six of the women had *Chlamydia trachomatis* infections, three had *Neisseria gonorrhoeae* infections, one had a *Trichomonas vaginalis* infection, one was shedding HSV-2 and only one participant was coinfected with *Neisseria gonorrhoeae and Chlamydia trachomatis*. At the time of sampling, none of the participants tested positive for *Treponema pallidum* or had yeast infections determined by examination of Gram stained slides.

**Table 1:** Demographic and clinical characteristics of the study population.

Clinical and laboratory findings	Optimal microbiota (N = 14) n (%)	Non-optimal Microbiota (N = 18) n (%)	P-value
Black race	14 (100)	18 (100)	
Median age in years (range)	18.5 (16–20)	19 (16–22)	p = 0.0001
*Chlamydia trachomatis* (PCR positive)	3 (21)	3 (17)	p > 0.9999
*Neisseria gonorrhoeae* (PCR positive)	3 (21)	0 (0)	p = 0.0734
*Trichomonas vaginalis* (PCR positive)	1 (7)	0 (0)	p = 0.4375
HSV-2 IgG positive	0 (0)	6 (0)	p = 0.0238
PSA positive	1 (7)	8 (44)	p = 0.0443
Using DMPA	3 (21)	2 (11)	p = 0.6313
Using Nur-Isterate	10 (71)	12 (67)	p > 0.9999
Using Implanon	1 (7)	4 (22)	p = 0.3547

PCR, polymerase chain reaction; PSA, prostate specific antigen; DMPA, depot medroxyprogesterone acetate. All participants were herpes simplex virus (HSV), *Mycoplasma genitalium* and *Treponema pallidum* PCR negative and did not have detectable yeast cells on Gram-stained vaginal smears. Mann-Whitney U test was used to compare continuous data and Fisher’s exact test was used for categorical data.

### *Lactobacillus* isolates from women with non-optimal induced greater inflammatory cytokine responses *in vitro* compared to isolates from women with optimal microbiota

**Figure 1:**
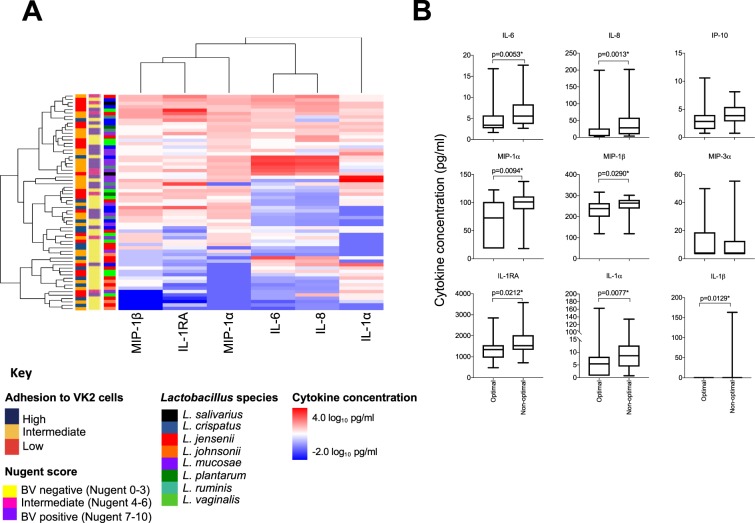
Cytokine production by vaginal epithelial (VK2) cells in response to vaginal *Lactobacillus* isolates. (**A**) Heatmap of log_10_-transformed concentrations of cytokines produced by VK2 cells stimulated with *Lactobacillus* isolates (n = 64) obtained from women with optimal (n = 36), intermediate (n = 8) and non-optimal microbiota (n = 20). *Lactobacillus* cultures were adjusted to 4.18 × 10^6^ colony forming units (CFU)/ml in antibiotic free keratinocyte serum free media then added to VK2 cell monolayers before being incubated for 24 hours at 37 °C with 5% CO_2_. Cytokine concentrations in the cell culture supernatants were measured using Luminex. Level of adhesion to VK2 cells, bacterial vaginosis (BV) status, and *Lactobacillus* species are also shown on the left side of the heatmap. (**B**) Inflammatory cytokine production in response to *Lactobacillus* isolates from women with optimal microbiota (Nugent 0–3; n = 36), including *L. crispatus* (n = 6); *L. jensenii* (n = 12), *L. johnsonii* (n = 5), *L. mucosae* (n = 4), *L. plantarum* (n = 1), *L. vaginalis* (n = 8), compared to women with non-optimal microbiota (Nugent 4–10; n = 28), including *L. crispatus* (n = 5), *L. jensenii* (n = 2), *L. mucosae* (n = 11), *L. plantarum* (n = 1), *L. ruminis* (n = 5), *L. salivarius* (n = 2) and *L. vaginalis* (n = 2). Data are shown as Tukey box plots. Boxes represent the interquartile ranges, lines within boxes represent medians and whiskers represent minimum and maximum values. P-values were adjusted for multiple comparisons using a false discovery rate step down procedure. *Adjusted p-values < 0.05 were considered statistically significant.

**Figure 2:**
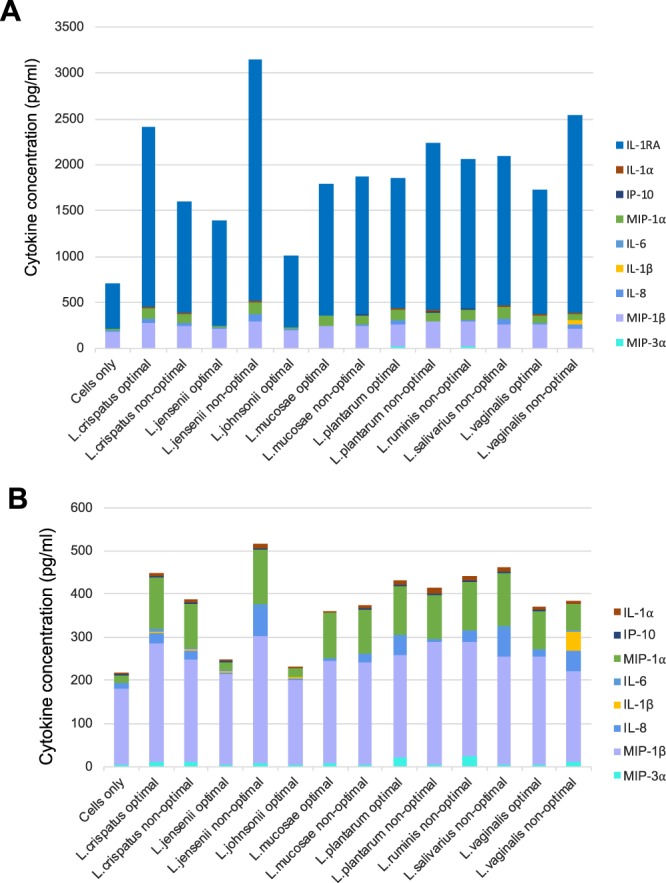
Inflammatory cytokine production by VK2 cells in response to different vaginal *Lactobacillus* species. *Lactobacillus* isolates, including *L. crispatus* (n = 11), *L. jensenii* (n = 14), *L. johnsonii* (n = 5), *L. mucosae* (n = 15), *L. plantarum* (n = 2), *L. ruminis* (n = 5), *L. salivarius* (n = 2) and *L. vaginalis* (n = 10), were adjusted to 4.18 × 10^6^ colony forming units (CFU)/ml in antibiotic free keratinocyte serum free media before being incubated with VK2 cells for 24 hours at 37 °C with 5% CO_2_. Cytokine concentrations were measured in the culture supernatants using Luminex. (**A**) Stacked bars showing median concentrations of each cytokine interleukin (IL)-1α, IL-1β, IL-6, IL-8, IFN-γ-inducible protein (IP)-10, macrophage inflammatory protein (MIP)-1α, MIP-1β, MIP-3α and regulatory IL-1 receptor antagonist (RA). (**B**) Stacked bars showing median concentrations of each cytokine excluding IL-1RA (IL-1α, IL-1β, IL-6, IL-8, IP-10, MIP-1α, MIP-1β and MIP-3α).

To determine whether inflammatory cytokine induction differed between lactobacilli obtained from women with optimal (BV negative; n = 14) and non-optimal (intermediate microbiota and BV positive (n = 18) microbiota, we stimulated vaginal epithelial (VK2) cells with 64 lactobacilli isolates in separate cultures and measured secreted interleukin (IL)-1α, IL-1β, IL-6, IL-8, IFN-γ-inducible protein (IP)-10, macrophage inflammatory protein (MIP)-1α, MIP-1β, MIP-3α and regulatory IL-1 receptor antagonist (RA) concentrations in cell culture supernatants using Luminex. Lactobacilli obtained from women with intermediate microbiota or BV induced greater inflammatory responses than isolates from women with optimal microbiota (Fig. 1A). IL-6 [adjusted (adj.) p = 0.020], IL-8 (adj. p = 0.011), IL-1α (adj. p = 0.020), MIP-1α (adj. p = 0.020), MIP-1β (adj. p = 0.040) and IL-1RA (adj. p = 0.030) production in response to isolates from women with non-optimal was significantly greater than lactobacilli from women with optimal microbiota (Fig. 1B). Similar responses were observed when evaluating inflammatory responses induced by isolates from women with intermediate microbiota and BV separately (data not shown). Of the different species evaluated, *L. jensenii* and *L. johnsonii* isolates tended to induce lower levels of cytokine production than the other isolates (Figs. 1A and 2). However, there were no significant differences in individual cytokines between species after adjusting for multiple comparisons and the level of within-species variation was high (Supplementary Fig. 1).

Logistic regression was used to evaluate the relationship between inflammatory responses to the *Lactobacillus* isolates and the BV status of the women, adjusting for possible confounders, including the presence of semen which may contain lactobacilli^29^, the use of contraceptives that may influence the microbial populations present^30^ and STIs which are associated with BV status. The relationships between BV status and IL-6 [β-coefficient: 2.71; 95% confidence interval (CI): 0.40–5.01; p = 0.021], IL-8 (β-coefficient: 1.44; 95% CI: 0.34–2.53; p = 0.010), MIP-1α (β-coefficient: 1.83; 95% CI: 0.06–3.60; p = 0.043) and IL-1RA (β-coefficient: 4.00; 95% CI: 0.39–7.62; p = 0.030) remained significant after adjusting for *Lactobacillus* species, semen contamination [determined by prostate specific antigen (PSA) measurement in the cervicovaginal secretions] and contraceptive use at the time of sample collection. Following adjustment for STI status, MIP-1α (β-coefficient: 1.90; 95% CI: 0.11–3.70; p = 0.038) and IL-8 (β-coefficient: 1.21; 95% CI: 0.13–2.29; p = 0.028) production remained significantly associated with the BV status of the women from whom the isolates were obtained.

To further confirm this observation, we compared inflammatory cytokine production in response to 16 different *Lactobacillus* isolates from women with optimal (n = 8) versus non-optimal (n = 8) microbiota. We again observed a clear difference in inflammatory cytokine production between the groups (Supplementary Fig. 2).

### *Lactobacillus* isolates suppressed vaginal epithelial cell inflammatory responses to *G. vaginalis*

**Figure 3:**
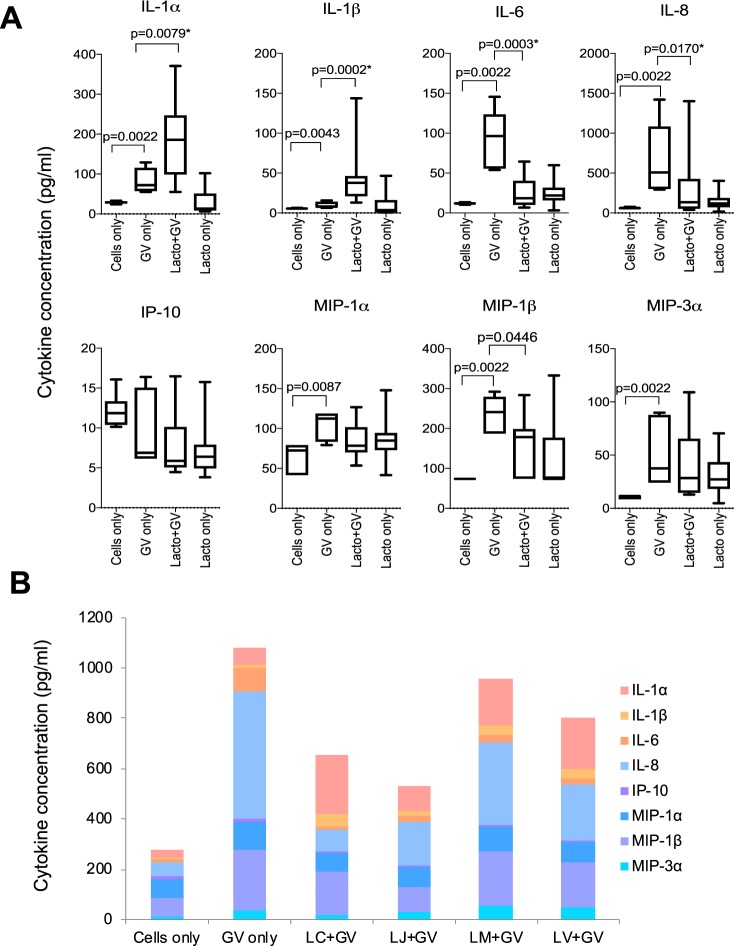
Cytokine production by VK2 cells in response to *Gardnerella vaginalis* in the presence or absence of clinical *Lactobacillus* isolates (n = 16). Immortalized VK2 cells were cultured to confluence and then treated with *Lactobacillus* isolates adjusted to 4.18 × 10^6^ colony forming units (CFU)/ml in antibiotic free keratinocyte serum free media before being incubated for 5 hours at 37 °C with 5% CO_2_. *G. vaginalis* cultures at a concentration of 1 × 10^7^ CFU/ml were then added and incubated for a further 20 hours. Cytokine concentrations were measured in the culture supernatants using Luminex. Mann Whitney U tests were used to compare cytokine responses and p-values were adjusted for multiple comparisons using a false discovery rate step down procedure. (**A**) Data are presented as Tukey box plots. Boxes represent the interquartile ranges, lines within boxes represent medians and whiskers represent minimum and maximum values. *Adjusted p-values < 0.05 were considered to be statistically significant. (**B**) Stacked bars showing the median concentrations of all pro-inflammatory cytokines and chemokines produced by VK2 cells in response to *G. vaginalis* in the presence or absence of different clinical *Lactobacillus* species, including *L. crispatus* (n = 4), *L. jensenii* (n = 4), *L. mucosae* (n = 4), and *L. vaginalis* (n = 4).

Previous studies have suggested that *Lactobacillus* species and their metabolites may suppress inflammatory responses to vaginal pathogens and pathobionts^27,28^. To investigate this, inflammatory cytokine production by VK2 cells in response to *G. vaginalis* was evaluated. Stimulating the cells with *G. vaginalis* induced production of IL-8 (adj. p = 0.005), IL-6 (adj. p = 0.005), MIP-1α (adj. p = 0.014), MIP-1β (adj. p = 0.005), MIP-3α (adj. p = 0.005) and IL-1α (adj. p = 0.005). Pretreating the cells with 16 lactobacilli in separate cultures suppressed production of IL-6 (adj. p = 0.002) and IL-8 (adj. p = 0.024) in response to *G. vaginalis*, while non-significant decreases in MIP-1α, MIP-1β, and MIP-3α were observed, with the concentrations of these mediators returning to levels that were comparable to *Lactobacillus* only cultures (Fig. 3A). However, pre-incubation with lactobacilli prior to *G. vaginalis* stimulation significantly increased the production of IL-1α (adj. p = 0.010) and IL-1β (adj. p = 0.002) relative to *G. vaginalis* alone or incubation with lactobacilli only. Overall, *L. jensenii* isolates suppressed cytokine responses to *G. vaginalis* to the greatest degree, followed by *L. crispatus, L. vaginalis* and *L. mucosae* (Fig. 3B). The *Lactobacillus* and *G. vaginalis* cultures showed no evidence of cytotoxicity to the VK2 cells after bacterial stimulations (Supplementary Fig. 3).

### *Lactobacillus* properties differed between women with optimal compared to non-optimal microbiota

**Figure 4:**
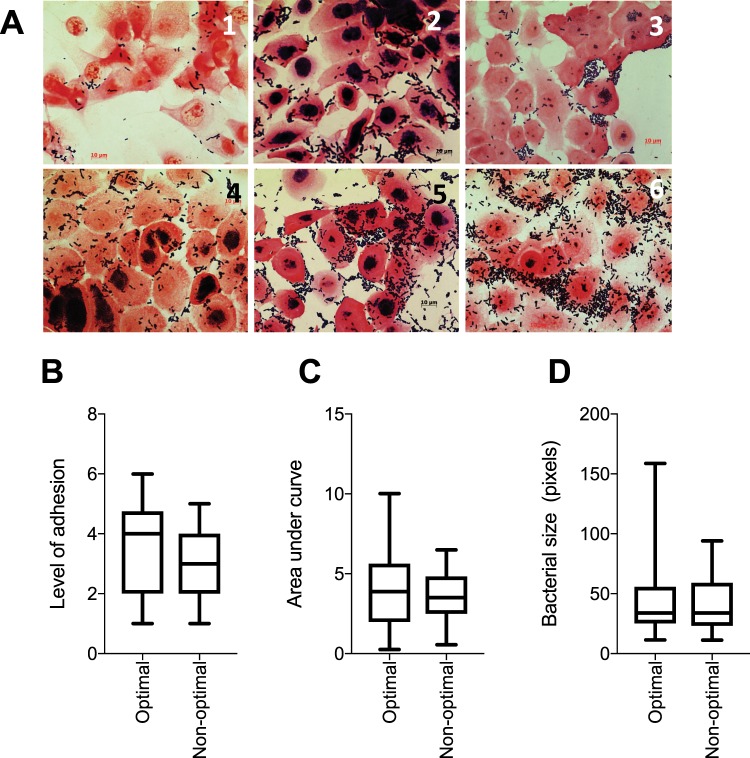
(**A**) Gram stained images of *Lactobacillus* adhesion to VK2 cells. *Lactobacillus* isolates (n = 64) were cultured and adjusted to 4.18 × 10^6^ colony forming units (CFU)/ml in antibiotic free keratinocyte serum free media before being added to VK2 cell monolayers in chamber slides and incubated for 2 hours at 37 °C with 5% CO_2_. Non-adherent bacteria were removed with sterile phosphate buffered saline (PBS) before the slides were Gram stained. Representative images of the Gram stained slides were collected and *Lactobacillus* isolates were ranked according to level of adhesion in ascending order from least adherent (**1**) to the most adherent (**6**). Level of adhesion to VK2 cells, growth rates and lengths of *Lactobacillus* isolates obtained from women with optimal [Nugent score: 0–3 (n = 36)] and non-optimal microbiota [Nugent score: 4–10 (n = 28)]. (**B**) Adhesion was determined by adding *Lactobacillus* cultures adjusted to 4.18 × 10^6^ colony forming units (CFU)/ml in antibiotic free keratinocyte serum free media to VK2 cell monolayers and incubating for 2 hours at 37 °C with 5% CO_2_. Non-adherent bacteria were removed with sterile phosphate buffered saline (PBS) before the slides were Gram stained. Each isolate was then scored according to level of adhesion (1–6) by two individuals blinded to the cytokine profiles of the isolates. (**C**) Growth rates were evaluated by measuring the optical densities at a wavelength of 600 nm, of *Lactobacillus* cultures initially adjusted to 4.18 × 10^6^ CFU/ml and incubated in de Man Rogosa and Sharpe (MRS) broth anaerobically for  24 hours. The areas under the curve were determined during the active phase of growth. (**D**) Relative bacterial size. Single colonies were picked from *Lactobacillus* cultures (n = 64) and smears were prepared on microscope slides and Gram-stained before taking images at 1,000x magnification. Bacterial length was determined from the images using Image J software. The mean of five measurements for each isolate was used for analysis. Boxes represent the interquartile ranges, lines within boxes represent medians and whiskers represent minimum and maximum values. P-values < 0.05 were considered statistically significant.

We next evaluated factors that could influence the immunoregulatory and protective properties of the lactobacilli by measuring D-lactate and L-lactate production (in bacterial culture and in VK2 cell co-culture), culture pH levels, lactobacilli growth rates, bacterial sizes and levels of adhesion to VK2 cells. To evaluate lactobacilli adhesion to epithelial cells, we co-cultured lactobacilli (n = 64) with VK2 cells for 2 hours, washed off unbound bacteria and qualitatively evaluated adhesion by Gram stain and microscopy. Lactobacilli were ranked according to the level of adhesion by scoring each image on a scale of 1 to 6 (Fig. 4A). It was found that lactobacilli adhesion to VK2 cells did not differ significantly between isolates from women with non-optimal versus those from women with optimal microbiota (Fig. 4B). Additionally, no significant differences were noted for *Lactobacillus* growth rates and sizes (Fig. 4C,D).

**Figure 5:**
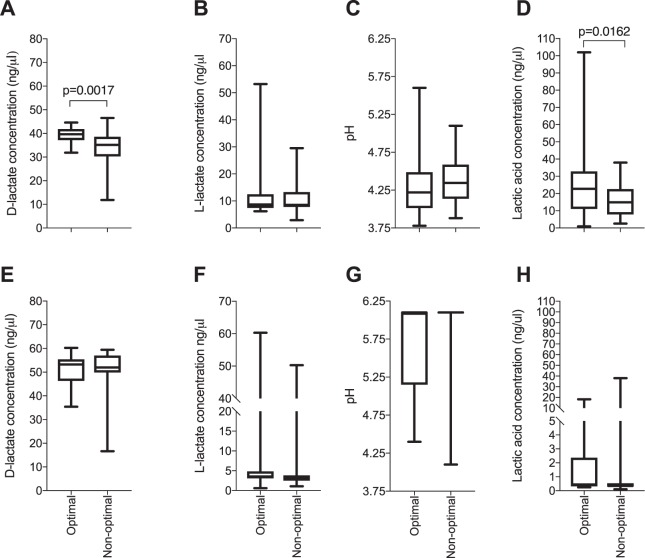
Comparison of D-lactate production, L-lactate production, culture acidification and total lactic acid production by clinical *Lactobacillus* isolates in bacterial cultures. (**A**–**D**) Characteristics in de Man Rogosa and Sharpe (MRS) culture; (**E**–**H**) Characteristics in *Lactobacillus*-VK2 cell co-cultures. *Lactobacillus* isolates obtained from women with optimal (n = 36) and non-optimal microbiota (n = 28) were cultured and adjusted to 4.18 × 10^6^ colony forming units (CFU)/ml in MRS broth and incubated anaerobically for 24 hours, or adjusted to 4.18 × 10^6^ CFU/ml in antibiotic free keratinocyte serum free media before being added to VK2 cell monolayers and incubated for 24 hours at 37 °C under 5% CO_2_. Supernatants were collected and the concentrations of D-lactate, L-lactate were determined using D-Lactate Colorimetric and Lactate Assay kits. Culture pH was measured using a pH meter in bacterial cultures and pH strips in cell co-cultures. Total lactic acid was calculated using the Henderson-Hasselbalch equation. Boxes represent the interquartile ranges, lines within boxes represent medians and whiskers represent minimum and maximum values. P-values < 0.05 were considered statistically significant.

Culture pH was measured using a pH meter, lactate was measured using ELISA and lactic acid concentrations were calculated using the Henderson-Hasselbach equation^31^. All of the lactobacilli isolates produced D-lactate in both bacterial and cell co-culture, but not all produced L-lactate. Total lactic acid strongly correlated with culture acidification in both cell co-culture (p < 0.0001; rho = −0.7912) and in bacterial culture (p < 0.0001; rho = −0.9034). Isolates from women with non-optimal microbiota produced significantly lower amounts of D-lactate (p = 0.0017) and lactic acid (p = 0.016) in bacterial culture compared to those from women with optimal microbiota (Fig. 5). However, neither D- nor L-lactate production differed significantly between species (Supplementary Fig. 4).

### Inflammatory cytokine production was associated with *Lactobacillus* adhesion to vaginal epithelial cells, D-lactate production and D-lactate dehydrogenase relative abundance

As lactic acid production by lactobacilli, as well as competitive exclusion of pathogens, may influence inflammatory responses, we next evaluated the relationships between inflammatory cytokines and lactate production and adhesion to vaginal epithelial cells. Overall, highly adherent isolates induced lower cytokine responses (Fig. 1A). *Lactobacillus* adhesion to VK2 cells correlated negatively with IL-6 (p = 0.0018, adj. p = 0.0162, rho = −0.3835), IL-8 (p = 0.0242, adj. p = 0.0726, rho = −0.2815), MIP-1α (p = 0.0233, adj. p = 0.0726, rho = −0.2833) and IL-1RA (p = 0.0355, adj. p = 0.080 rho = −0.2633).

To further investigate the impact of competitive binding of the lactobacilli to the VK2 cells, a variation of the cytokine assay was carried out in which unbound lactobacilli were then washed off with PBS before *G. vaginalis* was added. We found that washing off unbound lactobacilli reduced the level of inflammatory cytokine suppression (Supplementary Fig. 5), suggesting that the unbound lactobacilli also contribute to the immunoregulatory effect, perhaps through the production of metabolites such as lactic acid.

While L-lactate, culture pH, average bacterial length and growth rates were not associated with cytokine production, D-lactate production was negatively correlated with IL-6 concentrations in *Lactobacillus*/*G. vaginalis* co-cultures (rho = −0.6269; p = 0.0082; adj. p = 0.066), although this association was not upheld after adjusting for multiple comparisons. A negative trend towards an association between D-lactate and IL-8 production was also observed in these co-cultures (rho = −0.4971; p = 0.0501). To further evaluate this relationship, lactate dehydrogenase relative abundance in a subset of 44 isolates was assessed using proteomics. It was found that the production of D-lactate by lactobacilli isolates correlated positively with D-lactate dehydrogenase protein relative abundance (Spearman rho = 0.3457; p = 0.0215), while a trend towards a positive correlation was observed between L-lactate production and L-lactate dehydrogenase relative abundance (Spearman rho = 0.2754; p = 0.0700). Additionally, IL-6, IL-8, IP-10 and MIP-1α production by VK2 cells following incubation with the lactobacilli correlated inversely with isolate D-lactate dehydrogenase relative abundance, but not L-lactate dehydrogenase relative abundance (Supplementary Tables 1 and 2). Together these findings suggest that both D-lactate production and the direct interaction between the lactobacilli and epithelial cells play an important role in regulation of inflammatory responses by the lactobacilli.

## Discussion

Understanding the characteristics of *Lactobacillus* species and strains that may influence genital tract inflammatory cytokine responses and pathogen colonization is critical for the development of more effective treatment strategies for BV in order to move the field of HIV prevention in young women forward. In this study, we used *in vitro* systems to measure the concentrations of proinflammatory cytokines secreted by vaginal epithelial cells in response to 80 vaginal *Lactobacillus* isolates and *G. vaginalis*, a key BV-associated bacterial species. We found that *Lactobacillus* isolates from women with non-optimal microbiota (Nugent score: 4–10) were significantly more inflammatory than isolates from women with optimal microbiota (Nugent score: 0–3). It was further found that 16 *Lactobacillus* isolates were able to significantly suppress inflammatory responses to *G. vaginalis*. *Lactobacillus* isolates that induced greater inflammatory responses produced less D-lactate dehydrogenase and D-lactate than those that induced little inflammatory cytokine production in VK2 cells. Additionally, less adherent lactobacilli were more inflammatory than those that strongly adhered to vaginal epithelial cells.

In this study, large variation was observed between the inflammatory properties of vaginal *Lactobacillus* strains, even those of the same species. This highlights the need to understand not just species level changes, but also strain level variation in microbiome studies. Previous studies have shown that women with non-optimal microbiota have higher levels of genital inflammation compared to women with *Lactobacillus*-dominant microbiota^5,8^. However, to our knowledge, this study is the first to compare the inflammatory properties of lactobacilli isolated from women with non-optimal to those of women with optimal microbiota. Our findings suggest that the lactobacilli themselves may contribute to the inflammatory profile associated with non-optimal bacteria in the FGT, although, given the low relative abundance of lactobacilli in women with non-optimal microbiota^5,8,18^, this contribution may be minimal.

Although some lactobacilli induced inflammatory responses when cultured with vaginal epithelial cells in isolation, overall the lactobacilli significantly suppressed inflammatory responses to *G. vaginalis*. In this study, incubation of vaginal epithelial cells with *G. vaginalis* alone caused significant upregulation of multiple inflammatory cytokines (IL-6, IL-8, IL-1α, MIP-1α, MIP-1β and MIP-3α), while pre-incubation with lactobacilli resulted in significant downregulation of IL-6 and IL-8 and nonsignificant downregulation of each of the chemokines evaluated. These findings are similar to previous studies showing that *G. vaginalis* induces inflammatory responses both *in vitro* and *in vivo* and lactobacilli have immunoregulatory properties *in vitro* and are associated with low inflammatory cytokine levels *in vivo*^28,32,33^. Although the majority of inflammatory cytokines and chemokines were lower following pre-incubation with lactobacilli prior to incubation with *G. vaginalis*, we found that IL-1α and IL-1β production was significantly higher compared to *G. vaginalis* and *Lactobacillus* only cultures. This suggests that co-culture of vaginal epithelial cells with both lactobacilli and *G. vaginalis* had an additive effect on the IL-1 pathway and that the production of the other cytokines assessed may be regulated through alternative pathways. The IL-1 pathway is regulated both post-transcriptionally and translationally and involves more complex regulated checkpoints compared to other cytokine systems^34,35^, which may explain the difference in expression of IL-1 compared to other cytokines observed in this study. Nevertheless, the fact that the majority of cytokines were suppressed by lactobacilli and cumulative median cytokine levels were lower following pre-incubation with lactobacilli compared to *G. vaginalis* only cultures suggests that lactobacilli may decrease HIV acquisition risk by reducing inflammatory cytokine production in the FGT. The mechanisms by which *G. vaginalis* induces inflammatory responses are not fully understood, however studies have shown that *G. vaginalis* produces a toxin, vaginolysin, that is cytolytic to host cells^36^. Damaged tissue releases danger associated molecular patterns which activate pattern recognition receptors to induce a pro-inflammatory response^37^. It has further been reported that vaginolysin treatment of HeLa cells *in vitro* activates the p38 mitogen activated protein kinase pathway and increases IL-8 production^36^. Recently it has been shown that *L. crispatus* is able to suppress vaginolysin expression by *G. vaginalis*^38^, providing a possible mechanism for the reduced production of some of the cytokines observed in this study.

In order to evaluate possible underlying mechanisms for the increased inflammatory response to lactobacilli from women with non-optimal microbiota that was observed, we assessed a range of properties of the lactobacilli that may influence inflammatory cytokine induction, including D-lactate, L-lactate and lactic acid production, lactate dehydrogenase relative abundance, culture acidification, growth rates, adhesion to vaginal epithelial cells and *Lactobacillus* sizes. All isolates produced D-lactate, while only some produced L-lactate, and, similar to inflammatory responses, there was a large amount of variation in these properties between strains, even within species. Additionally, isolates from women with non-optimal microbiota produced significantly lower amounts of D-lactate and lactic acid. A previous study similarly found that, while there were no differences in D-lactic acid production between different *Lactobacillus* species isolated from the FGT, isolates from women with BV produced lower amounts compared to those of women with optimal microbiota^39^. This suggests that the amount of vaginal lactic acid is largely dependent on the particular *Lactobacillus* species or strains that predominate, as previously suggested^40^. Additionally, as lactic acid contributes to maintaining a pH below 4.5 in the FGT which hinders the growth of BV-associated bacteria and pathogens^25,31^, the lower amounts of lactic acid produced by isolates from women with non-optimal microbiota may reflect their inability to protect against colonization by potentially pathogenic bacteria.

D-lactate and D-lactate dehydrogenase production by *Lactobacillus* isolates were inversely associated with cytokine production, supporting the results of previous studies demonstrating that lactic acid can have anti-inflammatory effects *in vitro*^27^. Additionally, adhesion of lactobacilli to vaginal epithelial cells was inversely associated with cytokine responses, suggesting that direct interaction between the isolates and vaginal epithelial cells is important for immunoregulation. Previous studies have suggested that the peptidoglycan cell wall, the proteins present in the cell wall, as well as the cell membrane, may influence the immunomodulatory properties of *Lactobacillus* species^41^. Thus, differential adhesion capabilities may reflect differences in cell wall and membrane properties. It was found that removing unbound lactobacilli and the culture supernatant prior to addition of *G. vaginalis* reduced the level of suppression of inflammatory responses, although cytokine downregulation was still observed. The reduction in cytokine secretion observed in the *Lactobacillus/G.vaginalis* co-cultures may thus be due to competitive exclusion of *G. vaginalis* interaction with the vaginal epithelial cells as well as an effect of metabolites being secreted by the lactobacilli. It has been shown previously that lactobacilli were able to reduce *G. vaginalis* adhesion to the mucosal epithelium by approximately 60%^42^, and that *G. vaginalis* was displaced from vaginal cells by lactobacilli^43^. Previous studies have additionally shown that vaginal lactobacilli reduce the expression of toll-like receptor (TLR)-4, which recognizes lipopolysaccharide (LPS) in the cell walls of Gram negative bacteria^44–46^. Although it seems that *G. vaginalis* does not express LPS, lactobacilli may suppress cytokine responses by reducing the production of other pattern recognition receptors^47^. Additionally, studies using cell lines have found that lactobacilli interfere with the nuclear factor kappa light-chain-enhancer of activated B cells (NF-kB) pathway, reducing inflammatory responses *in vitro*^48^. Contrary to these findings, other studies have observed increased immune activation via the TLR, NF-κB and p38 MAP kinase signalling pathways by some *Lactobacillus* strains, suggesting that immunomodulation by lactobacilli and the possible underlying mechanisms are highly strain-specific^49,50^.

Although this study provides valuable information about the inflammatory properties of clinical *Lactobacillus* species and strains, a limitation is that an *in vitro* model including a transformed primary cell line was utilized to evaluate the characteristics of *Lactobacillus* isolates and this environment does not perfectly mimic *in vivo* conditions. Another limitation is that the study was not powered to examine differences in the inflammatory nature within individual species.

In summary, these data show that non-*iners* vaginal *Lactobacillus* isolates induced varying levels of inflammatory cytokine production when cultured with vaginal epithelial cells, while isolates from women with non-optimal microbiota were more inflammatory *in vitro* than isolates from women with optimal microbiota. However, pre-incubation of vaginal epithelial cells with lactobacilli prior to the addition of *G. vaginalis*, resulted in decreases in the majority of the cytokines assessed. This study suggests that the properties of the particular *Lactobacillus* strains present in the FGT (including lactic acid production and inflammatory nature) may influence the ability of non-optimal bacteria to colonize this compartment and shows that the immunomodulatory mechanisms of lactobacilli are multifactorial. The findings of this study are relevant to biotherapeutic development, suggesting that it is critical to obtain *Lactobacillus* isolates from women with optimal microbiota and to fully characterize the inflammatory properties of potential vaginal probiotics.

## Methods

### Study design and sample selection

We carried out a cross-sectional observational study to assess the immunoregulatory properties of lactobacilli isolated from cervicovaginal secretions collected from young women who participated in the Women’s Initiative in Sexual Health (WISH) study in Cape Town, South Africa^8^. The parent study cohort comprised 149 women (16–22 years) and the present sub-study included 32 women. Demographic data was collected from the women by questionnaire and vulvovaginal swabs were collected for detection of STIs by nucleic acid amplification tests (HSV-1, HSV-2, *Mycoplasma genitalium, Trichomonas vaginalis, Neisseria gonorrhoeae*, *Chlamydia trachomatis* and *Treponema pallidum*), while candidiasis and BV were assessed by Gram stain, microscopy and Nugent scoring. Women with BV had Nugent scores ≥7; women with intermediate microbiota had Nugent scores between 4–6; women who were BV negative had scores between 0–3. Cervicovaginal secretions were also collected using menstrual cups (Softcup, Evofem Inc, San Diego, CA,) and 115 lactobacilli were isolated from the vaginal fluid and stored in 60% glycerol. From these, 80 isolates (44 from BV negative women, 28 from BV positive women, and 8 from women with intermediate microbiota) were selected for detailed characterization.

### Bacterial isolation

Lactobacilli were isolated from cervicovaginal secretions by culturing in de Man Rogosa and Sharpe (MRS) broth for 48 hours at 37 °C under anaerobic conditions. The cultures were streaked onto MRS agar plates under the same culture conditions, single colonies were picked and then pre-screened microscopically by Gram staining. Matrix Assisted Laser Desorption Ionization Time of Flight (MALDI-TOF), a technique that measures the unique protein profile of an organism, was conducted at the University of the Western Cape to identify the bacteria to species level. Bacterial growth rates in MRS broth under anaerobic conditions were determined by measuring the optical densities, at a wavelength of 600 nm, of lactobacilli cultures initially adjusted to 4.18 × 10^6^ CFU/ml at six time-points for  24 hours.

### Vaginal epithelial cell stimulation and measurement of cytokine concentrations

Vaginal epithelial cells (VK2/E6E7 ATCC CRL-2616), that closely resemble the tissue of origin, were maintained in complete keratinocyte serum free media (KSFM) supplemented with 0.4 mM calcium chloride, 0.05 mg/ml of bovine pituitary extract, 0.1 ng/ml human recombinant epithelial growth factor and 50 U/ml penicillin and 50 U/ml streptomycin (Sigma-Aldrich, St. Louis, Missouri) as described previously. The VK2 cells were seeded into 24-well tissue culture plates, incubated at 37 °C in the presence of 5% carbon dioxide and grown to confluency.

Sixteen lactobacilli comprising 4 *L. crispatus*, 4 *L. jensenii*, 4 *L. mucosae* and 4 *L. vaginalis* were each adjusted to 4.18 × 10^6^ CFU/ml in antibiotic-free KSFM and added to VK2-cell monolayers in culture and incubated for 5 hours. *G. vaginalis* ATCC 14018 cultures standardized to 1 × 10^7^ CFU/ml in antibiotic-free KSFM were then added to the cells and plates were incubated for a further 20 hours at 37 °C in the presence of 5% carbon dioxide^28^. Supernatants were collected for cytokine and lactate measurement. IL-6, IL-8, IL-1α, IL-1β, IP-10, MIP-3α, MIP-1α and MIP-1β concentrations were measured using a Magnetic Luminex Screening Assay kit (R&D, Minneapolis, Minnesota). We used a Bio-Plex Suspension Array Reader to collect data and a 5-parameter logistic regression to calculate cytokine concentrations from the standard curves using BIO-plex manager software (version 4; Bio-Rad Laboratories Inc, Hercules, California). Cytokine concentrations below the detectable limit were assigned the value of half the lowest recorded concentration of that cytokine. To confirm VK2 cell viability following bacterial stimulation, we used the Trypan blue exclusion assay. Viable and dead cells were counted using a light microscope. VK2 cell viability was expressed as a percentage of viable cells in relation to the total number of cells counted.

Thereafter, 64 lactobacilli adjusted to 4.18 × 10^6^ CFU/ml in antibiotic-free KSFM were used to stimulate VK2 cells for 24 hours at 37 °C in the presence of 5% carbon dioxide. Production of IL-6, IL-8, IL-1α, IL-1β, IP-10, MIP-3α, MIP-1α, MIP-1β and IL-1RA were measured as described above. The quality of cytokine data was assessed using Spearman Rank test, with technical replicates correlating strongly for all cytokines assessed (p < 0.001 for all; Supplementary Table 3).

### D- and L-lactate production by *Lactobacillus* isolates and pH changes

D- and L-lactate concentrations were measured in both *Lactobacillus* MRS culture and in *Lactobacillus*-VK2 co-culture supernatants. For the evaluation of lactate production in MRS, lactobacilli were adjusted to 4.18 × 10^6^ CFU/ml before being incubated for 24 hours under anaerobic conditions. For lactate measurement in co-cultures, supernatants were collected from *Lactobacillus*-VK2 co-cultures as described above. The concentrations of D-and L-lactate were determined in duplicate using D-Lactate Colorimetric and Lactate Assay kits (Sigma-Aldrich, St Louis, Missouri) according to the manufacturer’s protocol. Optical densities were measured at 450 nm for D-lactate and 570 nm for L-lactate and values were converted to ng/μl against standard curve values, according to manufacturer’s instructions. Culture pH in the *Lactobacillus*-VK2 co-culture systems was measured using pH strips (Macherey-Nagel, GmbH and Co., Duren, Germany) and a pH meter was used for *Lactobacillus* culture supernatants.

### *Lactobacillus* adhesion to vaginal epithelial cells

Monolayers of VK2 cells were cultured to confluency in 8-well chamber slides (Thermo Fisher Scientific Inc., Waltham, Massachusetts). *Lactobacillus* isolates were cultured in MRS broth, adjusted to 4.18 × 10^6^ CFU/ml in antibiotic-free KSFM and then added to the cells before being incubated for 2 hours at 37 °C with 5% carbon dioxide. The cell culture medium was removed from the wells and each well was washed 3 times with 1 ml PBS. The chambers were removed as per manufacturer’s instructions before each slide was heat-fixed. The slides were Gram-stained and representative images were collected (Leica ICC50 HD, Leica Microsystems, Wetzlar, Germany). Each isolate was then scored according to level of adhesion (1–6) by two individuals blinded to the cytokine profiles of the isolates. Gram stained images were also taken from single lactobacilli colonies smeared onto slides from MRS agar plates. Relative bacterial size was measured from images of the Gram-stained slides taken at a 1,000x magnification using Image J software. The mean of five measurements for each isolate was used for analysis.

### Measurement of lactate dehydrogenase expression by *Lactobacillus* isolates using mass spectrometry

To further evaluate the role of lactic acid in modulating the inflammatory properties of the lactobacilli, lactate dehydrogenase relative abundance was evaluated using proteomics analysis of 44 of the isolates (7 *L. crispatus*, 13 *L. jensenii*, 5 *L. johnsonii*, 9 *L. mucosae*, 1 *L. plantarum*, 4 *L. ruminis*, 2 *L. salivarus* and 3 *L. vaginalis*). The *Lactobacillus* isolates were adjusted to 4.18 × 10^6^ CFU/ml in MRS and incubated for 24 hours under anaerobic conditions. Following incubation, the cultures were centrifuged and the pellets washed 3x with PBS. Protein was extracted by resuspending the pellets in 100 mM triethylammonium bicarbonate (TEAB; Sigma T7408) 4% sodium dodecyl sulfate (SDS; Sigma 71736), sonication and incubation at 95 °C for 10 min. Nucleic acids were degraded using benzonase nuclease (Sigma E1014) and samples were clarified by centrifugation at 10 000 × g for 10 min. Quantification was performed using the Quanti-Pro BCA assay kit (Sigma QPBCA). HILIC beads (ReSyn Biosciences, HLC010) were washed with 250 μl wash buffer (15% ACN, 100 mM Ammonium acetate (Sigma 14267) pH 4.5). The beads were then resuspended in loading buffer (30% ACN, 200 mM Ammonium acetate pH 4.5). A total of 50 μg of protein from each sample was transferred to a protein LoBind plate (Merck, 0030504.100). Protein was reduced with tris (2-carboxyethyl) phosphine (Sigma 646547) and alkylated with methylmethanethiosulphonate (MMTS; Sigma 208795). HILIC magnetic beads were added at an equal volume to that of the sample and a ratio of 5:1 total protein and incubated on the shaker at 900 rpm for 30 min. After binding, the beads were washed four times with 95% ACN. Protein was digested by incubation with trypsin for four hours and the supernatant containing peptides was removed and dried down. Liquid chromatography tandem mass spectrometry analysis (LC-MS/MS) was conducted with a Q-Exactive quadrupole-Orbitrap (Thermo Fisher Scientific, USA) coupled with a Dionex UltiMate 3000 nano-HPLC system. Raw files were processed with MaxQuant version 1.5.7.4 against a database including the *Lactobacillus* genus and common contaminants.

### Statistical analysis

Data was analysed using STATA Version 12 (StataCorp, College Station, Texas), GraphPad Prism version 7 (GraphPad software, San Diego, California) and R Version 1.1.447 (The R Foundation for Statistical Computing, Vienna, Austria). Unsupervised hierarchical clustering was used to evaluate overall cytokine production in response to the isolates. Mann-Whitney U test was used for unmatched comparisons and a false discovery rate step-down procedure was used to adjust p-values for multiple comparisons. Spearman Rank test was used to test for correlations. Multivariate linear and logistic regression analyses were used to adjust for possible confounders.

### Ethics approval and participant consent

Ethical approval to conduct the parent study was obtained from the University of Cape Town (UCT) human research ethics committee (UCT HREC: 267/2013). The current sub-study was approved by the UCT human research ethics committee (UCT HREC: 551/2016) and all experiments were performed in accordance with relevant guidelines and regulations. Women older than 18 years provided written informed consent, while those who were 16–17 years old provided assent and written informed consent was obtained from their parents or legal guardians.

## Supplementary information

Supplementary information

## Data Availability

The datasets generated during and/or analysed during the current study are available from the corresponding author on reasonable request.
