# Supplementary material for: Inflammatory and antimicrobial properties differ between vaginal Lactobacillus isolates from South African women with non-optimal versus optimal microbiota
Source: Sci Rep. 2020 Apr 10;10:6196. doi: 10.1038/s41598-020-62184-8 (PMC7148372; doi:10.1038/s41598-020-62184-8)
Supplement: Supplementary file 1 — Supplementary information [file 41598_2020_62184_MOESM1_ESM.pdf]

Inflammatory and antimicrobial properties differ between vaginal *Lactobacillus* isolates from South African women with non-optimal versus optimal microbiota

Authors: Monalisa T. Manhanzva[1], Andrea G. Abrahams[1], Hoyam Gamieldien[1], Remy Froissart[2], Heather Jaspan[1,3], Shameem Z. Jaumdally[1], Shaun L. Barnabas[1], Smritee Dabee[1], Linda G. Bekker[1,4], Glenda Gray[5,6], Jo-Ann S. Passmore[1,7,8], Lindi Masson[1,7,9]

Affiliations: [1]Institute of Infectious Disease and Molecular Medicine (IDM), University of Cape Town, Cape Town, South Africa; [2]UMR 5290 MIVEGEC, French National Centre for Scientific Research (CNRS), Montpellier, France [3]Seattle Children's Research Institute, University of Washington, Seattle, Washington, USA; [4]Desmond Tutu HIV Centre, University of Cape Town, South Africa; [5]Perinatal HIV Research Unit, University of the Witwatersrand, Johannesburg, South Africa; [6]South African Medical Research Council, Cape Town, South Africa; [7]Centre for the AIDS Programme of Research in South Africa (CAPRISA), Durban, South Africa; [8]National Health Laboratory Service, Cape Town, South Africa; [9]Disease Elimination Program, Life Sciences Discipline, Burnet Institute, Melbourne, Australia

Correspondence: L. Masson (email: L.Masson@uct.ac.za)

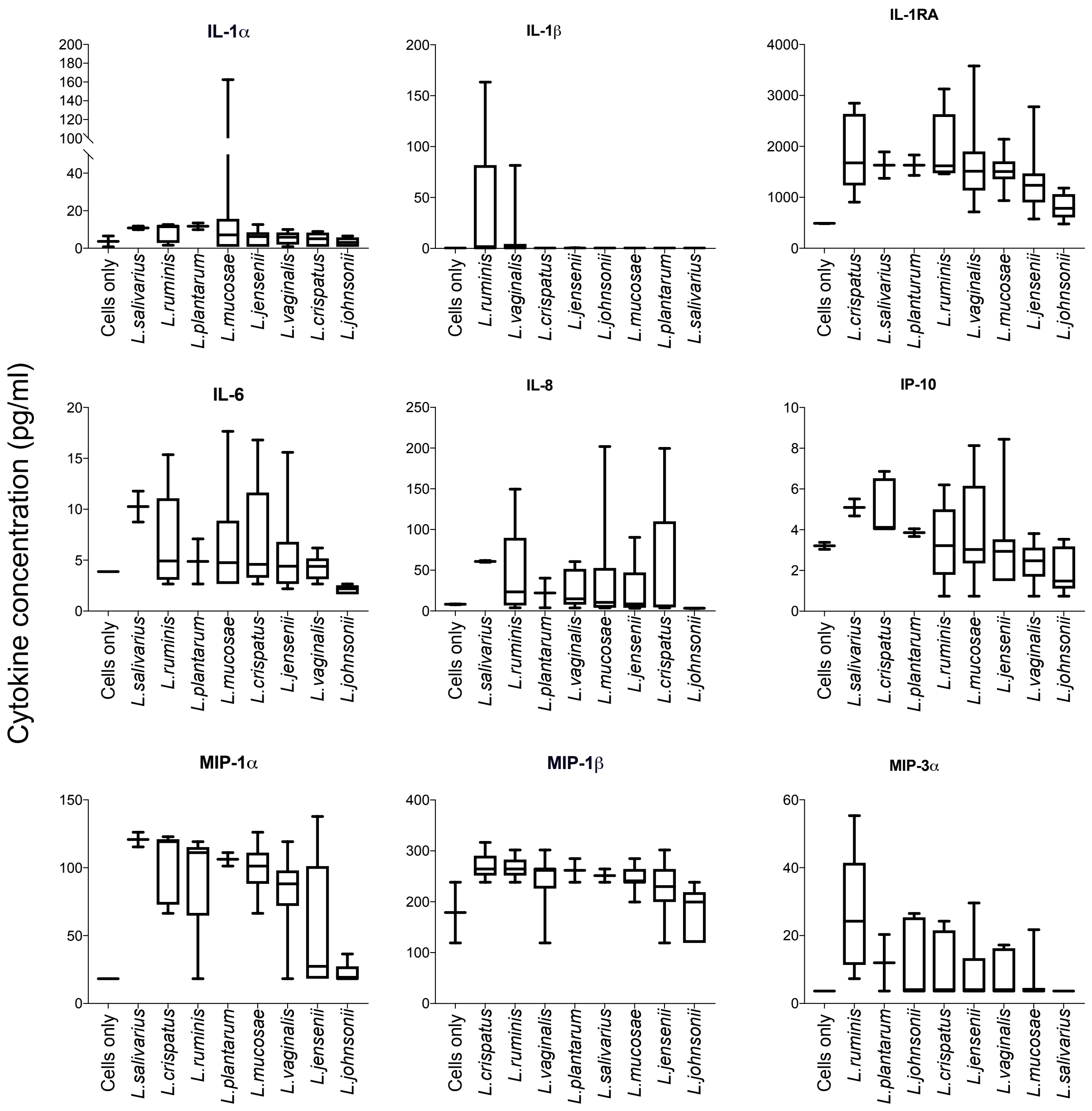

Supplementary Figure 1 Cytokine production by vaginal epithelial (VK2) cells in response to vaginal *Lactobacillus* isolates stratified by species. *Lactobacillus* cultures were adjusted to 4.18×10^6 colony forming units (CFU)/ml in antibiotic free keratinocyte serum free media then added to VK2 cell monolayers before being incubated for 24 hours at 37°C with 5% CO_2. Cytokine concentrations in the cell culture supernatants were measured using Luminex. *Lactobacillus* isolates are grouped by species, including *L. crispatus* (n=11); *L. jensenii* (n=14), *L. johnsonii* (n=5), *L. mucosae* (n=15), *L. plantarum* (n=2), *L. ruminis* (n=5), *L. salivarius* (n=2) and *L. vaginalis* (n=10). Data are shown as Tukey box plots. Boxes represent the interquartile ranges, lines within boxes represent medians and whiskers represent minimum and maximum values. No statistically significant differences ($p<0.05$) were observed.

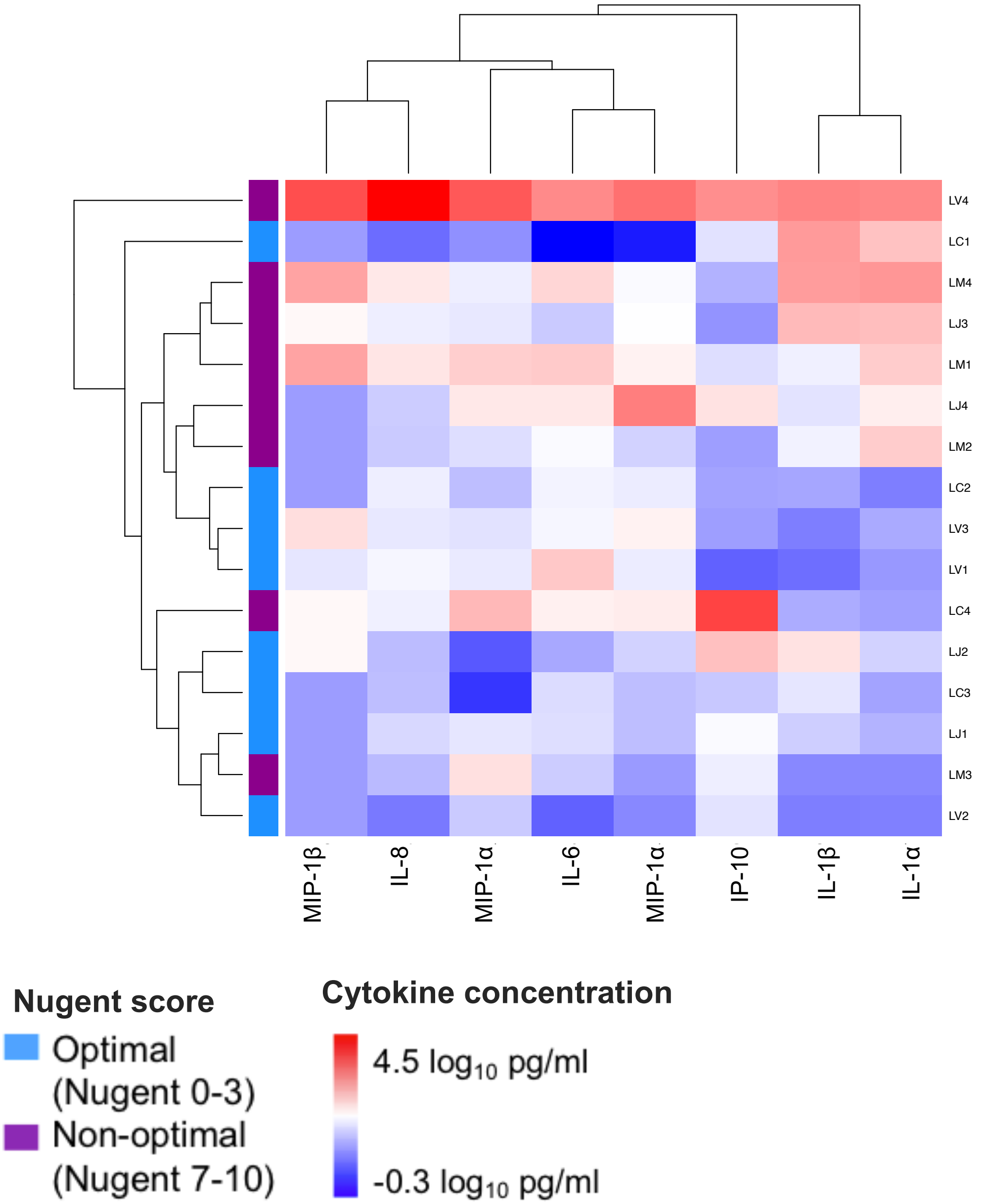

Supplementary Figure 2 Cytokine production by vaginal epithelial (VK2) cells in response to vaginal *Lactobacillus* isolates. Heatmap of $\log_{10}$-transformed concentrations of cytokines produced by VK2 cells stimulated with *Lactobacillus* isolates (n=16) obtained from women with optimal (n=8) and non-optimal microbiota (n=8). *Lactobacillus* cultures were adjusted to 4.18×10^6 colony forming units (CFU)/ml in antibiotic free keratinocyte serum free media then added to VK2 cell monolayers before being incubated for 24 hours at 37°C with 5% CO_2. Cytokine concentrations in the cell culture supernatants were measured using Luminex. Bacterial vaginosis (BV) status is shown on the left side of the heatmap.

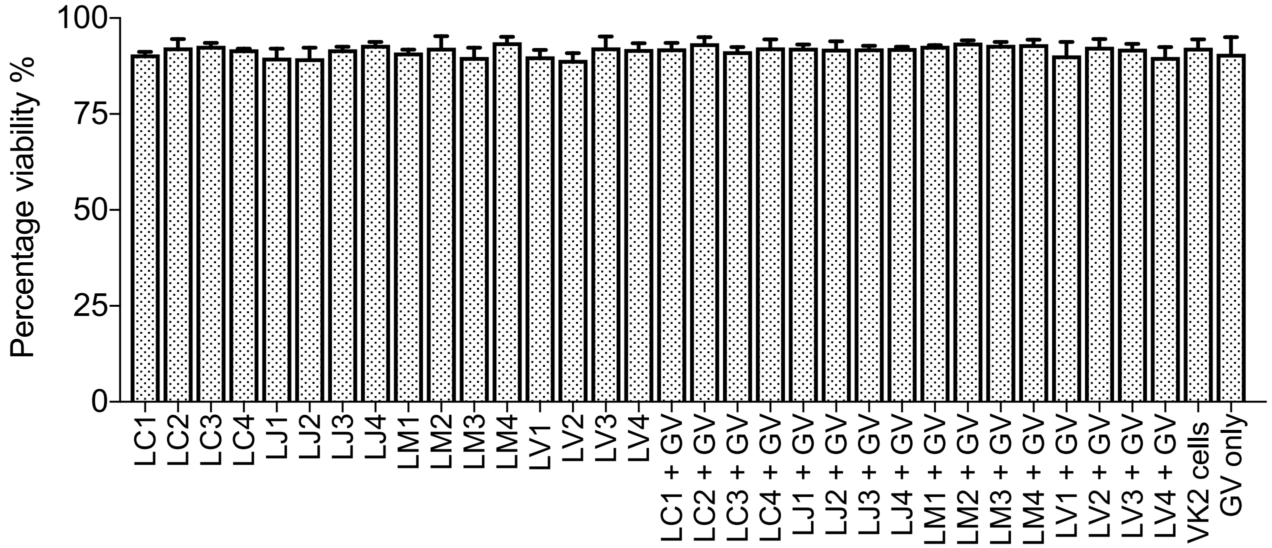

Supplementary Figure 3 Viability of vaginal epithelial cells incubated with lactobacilli and *Gardnerella vaginalis*. Immortalized VK2 cells were cultured to confluence and then treated with *Lactobacillus* isolates adjusted to 4.18 x 10^6 colony forming units (CFU)/ml in antibiotic free keratinocyte serum free media before being incubated for 5 hours at 37°C with 5% CO_2. *G. vaginalis* cultures at a concentration of 1 x 10^7 CFU/ml were then added and incubated for a further 20 hours. VK2 cell viability was assessed using the Trypan blue exclusion assay. Bars show the average of the percentage of viable cells in each culture, with errors bars showing ranges. LC: *L. crispatus*; LJ: *L. jensenii*; LM: *L. mucosae*; LV: *L. vaginalis*; GV: *G. vaginalis*.

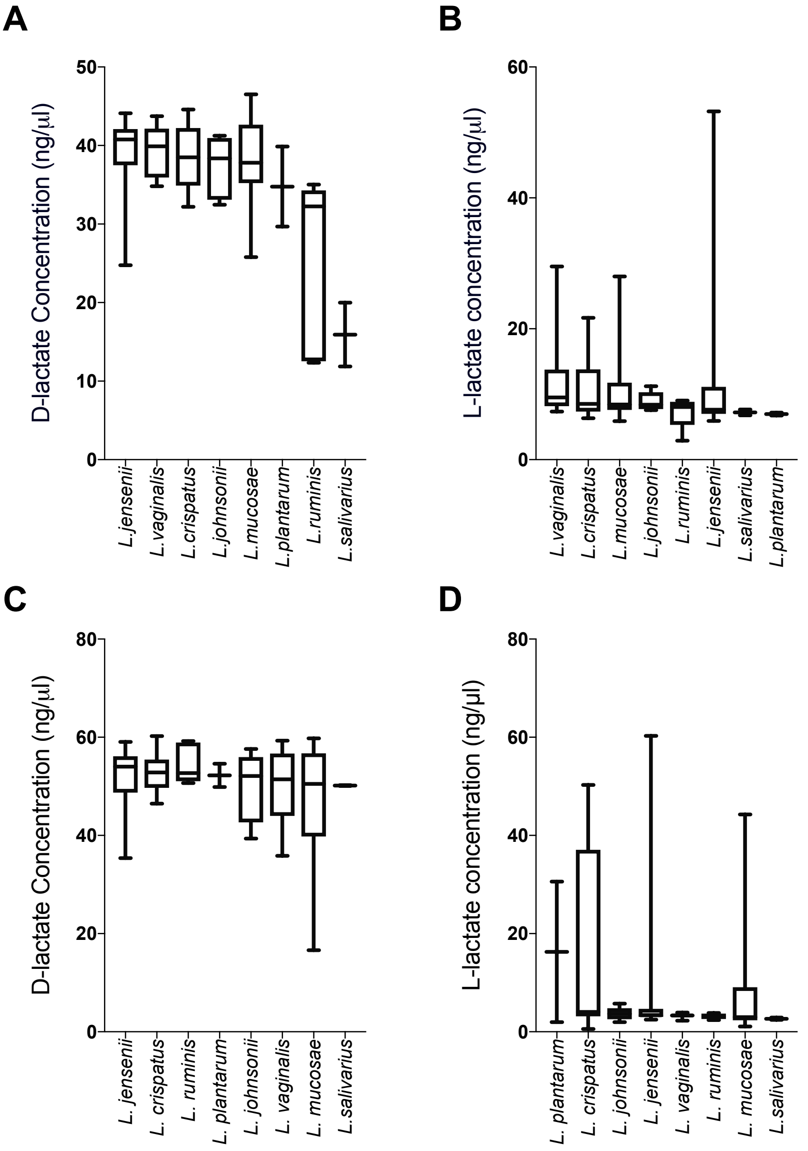

Supplementary Figure 4 Comparison of D-lactate production and L-lactate production between *Lactobacillus* species. (**A, B**) Lactate production in de Man Rogosa and Sharpe (MRS) culture. (**C, D**) Lactate production in *Lactobacillus*-VK2 cell co-cultures. *Lactobacillus* isolates were cultured and adjusted to 4.18 x10^6 colony forming units (CFU)/ml in MRS broth and incubated anaerobically for 24 hours or adjusted to 4.18 x10^6 CFU/ml in antibiotic free keratinocyte serum free media before being added to VK2 cell monolayers and incubated for 24 hours at 37°C under 5% CO_2. Supernatants were collected and the concentrations of D-lactate and L-lactate were determined using D-Lactate Colorimetric and Lactate Assay kits. Boxes represent the interquartile ranges, lines within boxes represent medians and whiskers represent minimum and maximum values. P-values <0.05 were considered statistically significant.

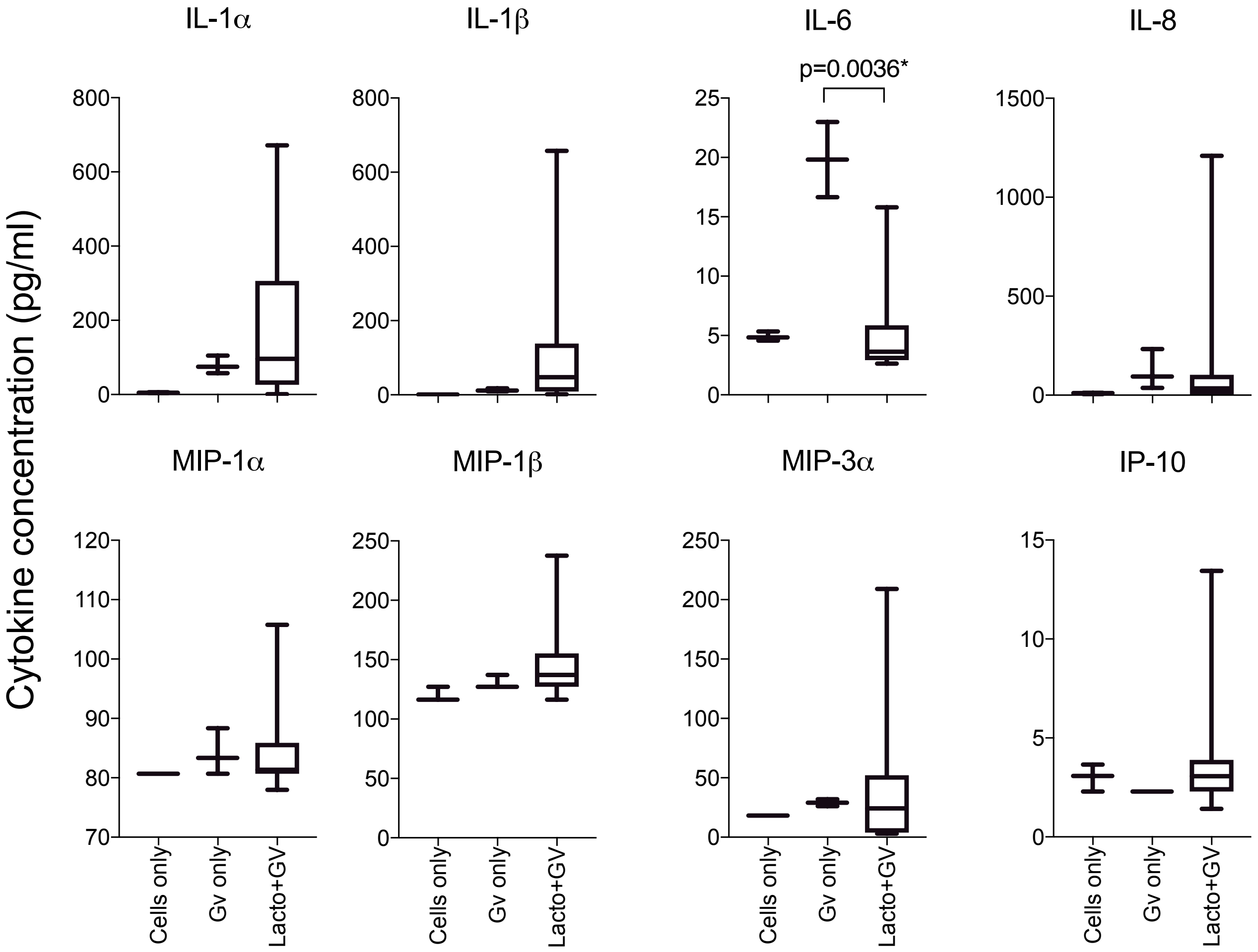

Supplementary Figure 5 Cytokine production by VK2 cells in response to *Gardnerella vaginalis* in the presence or absence of clinical *Lactobacillus* isolates (n=16). Immortalized VK2 cells were cultured to confluence and then treated with *Lactobacillus* isolates adjusted to 4.18 x 10^6 colony forming units (CFU)/ml in antibiotic free keratinocyte serum free media before being incubated for 5 hours at 37°C with 5% CO_2. Culture supernatants and unbound lactobacilli were then removed before *G. vaginalis* cultures at a concentration of 1 x 10^7 CFU/ml were added and incubated for a further 20 hours. Cytokine concentrations were measured in the culture supernatants using Luminex. Mann Whitney U tests were used to compare cytokine responses and p-values were adjusted for multiple comparisons using a false discovery rate step down procedure. Data are presented as Tukey box plots. Boxes represent the interquartile ranges, lines within boxes represent medians and whiskers represent minimum and maximum values. *Adjusted p-values <0.05 were considered to be statistically significant.

Supplementary Table 1. Spearman correlations between L-lactate dehydrogenase versus cytokine production

Cytokine	Spearman Rho	95% Confidence Interval	P-value
IL-1α	0.0390	-0.2693 to 0.3400	P=0.8017
IL-1β	0.0031	-0.3023 to 0.3079	P=0.9842
IL-6	-0.1074	-0.3995 to 0.2044	P=0.4876
IL-8	-0.1087	-0.4005 to 0.2032·	P=0.4826
IP-10	-0.1174	-0.4079 to 0.1947	P=0.4479
MIP-1α	0.0081	-0.2977 to 0.3125	P=0.9583
MIP-1β	0.0702	-0.2401 to 0.3674	P=0.6507
MIP-3α	0.2236	-0.0875 to 0.4949	P=0.1446
IL-1RA	0.1735	-0.1390 to 0.4545	P=0.2600

Supplementary Table 2. Spearman correlations between D-lactate dehydrogenase versus cytokine production

Cytokine	Spearman Rho	95% Confidence Interval	P-value
IL-1α	-0.1922	-0.4698 to 0.1199	P=0.2113
IL-1β	-0.0334	-0.3351 to 0.2745	P=0.8294
IL-6	-0.4766	-0.6825 to -0.2007	P=0.0011*
IL-8	-0.4341	-0.6528 to -0.1487	P=0.0032*
IP-10	-0.3722	-0.6083 to -0.0757	P=0.0128*
MIP-1α	-0.4362	-0.6542 to -0.1512	P=0.0031*
MIP-1β	-0.2579	-0.5219 to 0.0512	P=0.0910
MIP-3α	0.1202	-0.1919 to 0.4103	P=0.4369
IL-1RA	-0.2879	-0.5452 to 0.0189	P=0.0580

*Significant after false discovery rate adjustment for multiple comparisons

Supplementary Table 3. Cytokine data quality assessment of technical replicates

Cytokine	Spearman Rho	95% Confidence Interval	P-value	Samples with detectable concentrations (%)
IL-1α	0.9833	0.9650 to 0.9921	P<0.0001	96.9
IL-1β	0.9787	0.9555 to 0.9899	P<0.0001	95.3
IL-6	0.9611	0.9195 to 0.9814	P<0.0001	96.9
IL-8	0.9303	0.8763 to 0.9612	P<0.0001	93.8
IP-10	0.8189	0.6520 to 0.9101	P<0.0001	98.4
MIP-1α	0.6441	0.3717 to 0.8144	P<0.0001	90.6
MIP-1β	0.4504	0.1100 to 0.6962	P=0.0097	46.9
MIP-3α	0.9670	0.9314 to 0.9843	P<0.0001	95.3
